# CircPlant: An Integrated Tool for circRNA Detection and Functional Prediction in Plants

**DOI:** 10.1016/j.gpb.2020.10.001

**Published:** 2020-11-04

**Authors:** Peijing Zhang, Yongjing Liu, Hongjun Chen, Xianwen Meng, Jitong Xue, Kunsong Chen, Ming Chen

**Affiliations:** 1 Department of Bioinformatics, State Key Laboratory of Plant Physiology and Biochemistry, College of Life Sciences, Zhejiang University, Hangzhou 310058, China; 2 James D. Watson Institute of Genome Sciences, Zhejiang University, Hangzhou 310058, China; 3 Zhejiang Provincial Key Laboratory of Horticultural Plant Integrative Biology, Zhejiang University, Hangzhou 310058, China; 4 The State Agriculture Ministry Laboratory of Horticultural Plant Growth, Development and Quality Improvement, Zhejiang University, Hangzhou 310058, China

**Keywords:** Circular RNA, Plant, MicroRNA, RNA-seq, Identification

## Abstract

The recent discovery of **circular RNAs** (circRNAs) and characterization of their functional roles have opened a new avenue for understanding the biology of genomes. circRNAs have been implicated to play important roles in a variety of biological processes, but their precise functions remain largely elusive. Currently, a few approaches are available for novel circRNA prediction, but almost all these methods are intended for animal genomes. Considering that the major differences between the organization of **plant** and mammal genomes cannot be neglected, a plant-specific method is needed to enhance the validity of plant circRNA **identification**. In this study, we present CircPlant, an integrated tool for the exploration of plant circRNAs, potentially acting as competing endogenous RNAs (ceRNAs), and their potential functions. With the incorporation of several unique plant-specific criteria, CircPlant can accurately detect plant circRNAs from high-throughput **RNA-seq** data. Based on comparison tests on simulated and real RNA-seq datasets from *Arabidopsis thaliana* and *Oryza sativa*, we show that CircPlant outperforms all evaluated competing tools in both accuracy and efficiency. CircPlant is freely available at http://bis.zju.edu.cn/circplant.

## Introduction

Circular RNAs (circRNAs) constitute a large class of non-coding RNAs that were previously dismissed as molecular flukes or by-products of canonical RNA splicing. High-throughput sequencing technologies and bioinformatics approaches have revealed that circRNAs are endogenous, abundant, conserved across species, and widespread in eukaryotic transcriptomes [1], [2]. Emerging evidence suggests that circRNAs play important roles in diverse biological processes, including acting as competing endogenous RNAs (ceRNAs) or microRNA (miRNA) sponges, regulating alternative RNA splicing or transcription, and potentially enhancing host gene transcription [2]. Furthermore, circRNAs are involved in aging [3] and neural development [4], and their aberrant expression is associated with several diseases, including atherosclerotic vascular disease [5], colorectal and ovarian cancers [6], and esophageal squamous cell carcinoma [7]. As such, circRNAs could become promising biomarkers for disease diagnosis and prognosis. Although research of circRNAs has mainly focused on humans and animals, our knowledge and understanding on plant circRNAs has improved substantially as well.

Similar to animal circRNAs, plant circRNAs are conserved across different species, which have long flanking introns, are expressed at low levels with development/tissue-specific expression profiles, and may act as miRNA sponges or regulate the transcription of their parental genes [8], [9], [10], [11], [12], [13]. However, plant circRNAs have their own unique features. Studies of rice, soybeans, and cotton (*Gossypium*) have shown that plant exonic circRNAs are less likely to be generated from exons flanked by introns containing repetitive or reverse complementary sequences [11], [12], [13], [14]. Meanwhile, circRNAs have been observed to be paralog- and species-specific in soybean [11] and cotton [14]. For expression levels of exonic circRNAs and their parental genes, few have shown significant positive correlation, and no negatively-correlated pair has been found in rice [13]. A recent study in *Arabidopsis* has revealed that circRNA could bind to the DNA of its host gene through an R-loop to control linear alternative splicing, consequently regulating the transcription of its host gene [15]. As for biotic or abiotic stress conditions in plants, circRNAs are differentially expressed at specific time points/stages, which may act as important functional regulators involved in stress-specific biological processes in plants [16], [17], [18].

Given the implications of plant circRNAs in many biological processes, it is important to better understand their molecular mechanisms, characteristics, and functions. It is important to make improvements to our identification abilities. A key step is to improve our capabilities in identifying plant circRNAs. Many circRNA prediction tools have been developed (Table 1), which have been used to detect tens of thousands of circRNAs in human, mouse, fly, and other model organisms. Almost all the pipelines or programs are designed for mammals. However, the accuracy of predicting plant circRNAs is relatively low with available methods, potentially attributing to a high false positive rate or overlapping between identified plant circRNAs [9], [31], [32]. For example, in Ye et al.’s study [11], 10 of the 18 predicted exonic circRNAs were confirmed by sequencing PCR products amplified by divergent primers, but none of the 30 predicted non-exonic circRNAs was confirmed. Besides common canonical splicing signals in animals and humans, the majority of circRNAs (92.7%) in rice were flanked by non-GT/AG canonical splicing signals [33]. Considering the differences between plant and mammal genomes, a computational tool is urgently needed for improved identification of plant circRNAs.

**Table 1:** **Overview of circRNA detection tools**

**Tool**	***De novo***	**Mapper**	**Dependency**	**Check splice site**	**Language**	**Single-end**	**Latest release date**	**Refs.**
circRNA_finder	Yes	STAR	Awk, Samtools	GU-AG GC-AG AU-AC	Perl	No	2015	[3]
find_circ	Yes	Bowtie2	Pysam, Samtools	GU-AG	Python	No	2016	[19]
CIRCexplorer	Yes	Bowtie, TopHat	Cufflinks, Bedtools, pysam, Pandas, docopt	GU-AG GC-AG AU-AC	Python	Yes	2018	[20], [21]
Segemehl	Yes	Segemehl	Samtools	no	C	Yes	2015	[22]
CIRI	Yes	BWA-MEM	/	GU-AG	Perl	Yes	2017	[23], [24]
KNIFE	No	Bowtie, Bowtie2	Samtools, numpy, scipy, data.Table	no	Perl, Python, R	Yes	2017	[25]
MapSplice	No	Bowtie	Samtools	no	Python, C++	Yes	2016	[26]
UROBORUS	No	Bowtie, Bowtie2	TopHat, Samtools	no	Perl	Yes	2017	[27]
ACFS	Yes	BWA-MEM	/	full	Perl	Yes	2016	[28]
DCC/CircTest	No	STAR	Pysam, pandas, numpy, HTSeq	GU-AG	Python	Yes	2018	[29]
PcircRNA_finder	Yes	Bowtie, Bowtie2, STAR, Segemehl, TopHat	Pysam, docopt, Samtools, Bedtools	GU-AG GC-AG AU-AC	Perl, Python	No	2016	[30]
CircPlant	Yes	BWA-MEM	FASTA36, TAPIR, Bioperl	GU-AG GC-AG AU-AC	Perl	Yes	2018	–

*Note*: FASTA36 and TAPIR are required for the interaction prediction and network construction modules, while Bioperl is needed for the GO annotation module. The circRNA detection module in CircPlant has no dependencies.

According to previous results, circRNAs interact with miRNAs to regulate transcription and cellular pathways, act as miRNA sponges to naturally sequester and competitively inhibit the activity of miRNAs, or compete with miRNA-binding mRNAs [2]. circRNAs and those mRNAs with common miRNA target sites could form a complex interactive regulatory network, known as a ceRNA network [34]. Here, we present CircPlant, an integrated tool for plant circRNA detection and functional prediction, with circRNA-miRNA interactions, ceRNA pairs (or circRNA-miRNA-mRNA networks), and annotation of Gene Ontology (GO). Based on comprehensive comparisons applied to both simulated and real datasets, we have shown that CircPlant has the highest accuracy and precision among competing tools. More importantly, CircPlant enables huge reductions in runtime for detecting circRNAs from high-throughput sequencing data.

## Methods

**Figure 1:**
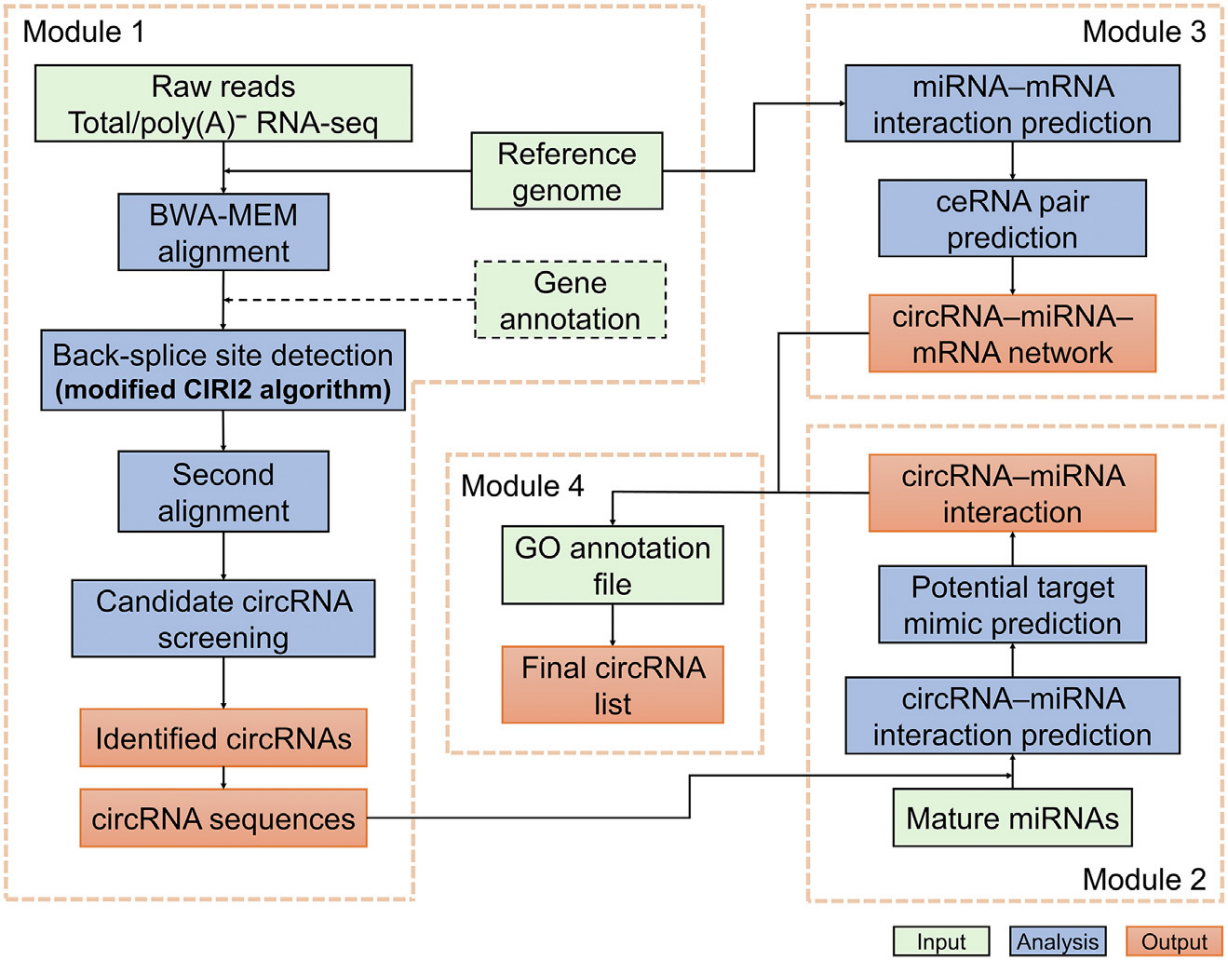
**Workflow of CircPlant** CircPlant consists of four modules, circRNA detection, circRNA-miRNA interaction prediction, circRNA-miRNA-mRNA network construction, and circRNA annotation.

As illustrated in Figure 1, the CircPlant tool consists of four modules (see details in File S1).

### Module 1: circRNA detection

Firstly, CircPlant uses a BWA-MEM algorithm [35] to map sequencing reads, and a plant-specifically modified CIRI2 [23], [24] algorithm is integrated to collect back-splice sites from rRNA-depleted total RNA or poly(A)-depleted RNA sequencing data. CIRI2 is a widely used computational detection method for circRNA identification, using an adapted maximum likelihood estimation based on multiple seed matching with remarkably balanced sensitivity, reliability, and duration relative to other methods [24], [31]. Several plant-specific factors are integrated into the modified CIRI2 (File S1), which account for the relatively small lengths of plant circRNAs, the high copy number of plant genes, and the non-canonical types of splicing signals [25], [33], [36], [37]. If gene annotation is provided, CIRI2 will combine the annotation file and plant-specific factors to screen candidate circRNA sites. Subsequently, these candidate sites are further screened through the second alignment. A pseudo reference genome (pseudoRef) is created with chiastic back-splice site flanking sequences. Raw reads are then mapped to it to check the back-splice sites [30]. Finally, full-length sequences of circRNAs are extracted, including isoforms possibly originated from the same back-splice sites [38]. For exonic circRNAs, which are generated from exons of a single protein-coding gene, CircPlant only extracts exon regions based on genome annotation. However, a few circRNAs were proven to contain sequences that cannot be found in mRNAs [10], [23], which may arise from the alternative splicing events within circRNAs [39]. These alternative splicing events in plants are not considered in the current algorithms, which should be addressed in future investigations.

### Module 2: circRNA–miRNA interaction prediction

To predict the interaction between circRNAs and miRNAs, all circRNA sequences are regarded as candidate miRNA targets. The target sequences that could be perfectly bound by mature miRNAs are identified by TargetFinder [40] with a score parameter less than three, and the potential target mimics are predicted by Tapir [41] with three filtering rules [42], [43] (File S1).

### Module 3: circRNA–miRNA–mRNA network construction

After obtaining the interactions between known miRNAs and mRNAs by TargetFinder, a hypergeometric test is applied to assess the reliability of the ceRNA pair prediction (File S1). The circRNA–miRNA–mRNA networks are then generated from ceRNA pairs, which can be exported as a file for visualization and modification in Cytoscape [44].

### Module 4: circRNA annotation

CircPlant applies the GO enrichment method to predict the potential biological functions of circRNAs based on their correlated ceRNA pairs. Given that members in a ceRNA pair are expected to have similar functions, the potential functions of a circRNA are assigned as enriched GO terms of all associating mRNAs in ceRNA pairs.

## Results

### The characteristics and methodological aspects of CircPlant

**Figure 2:**
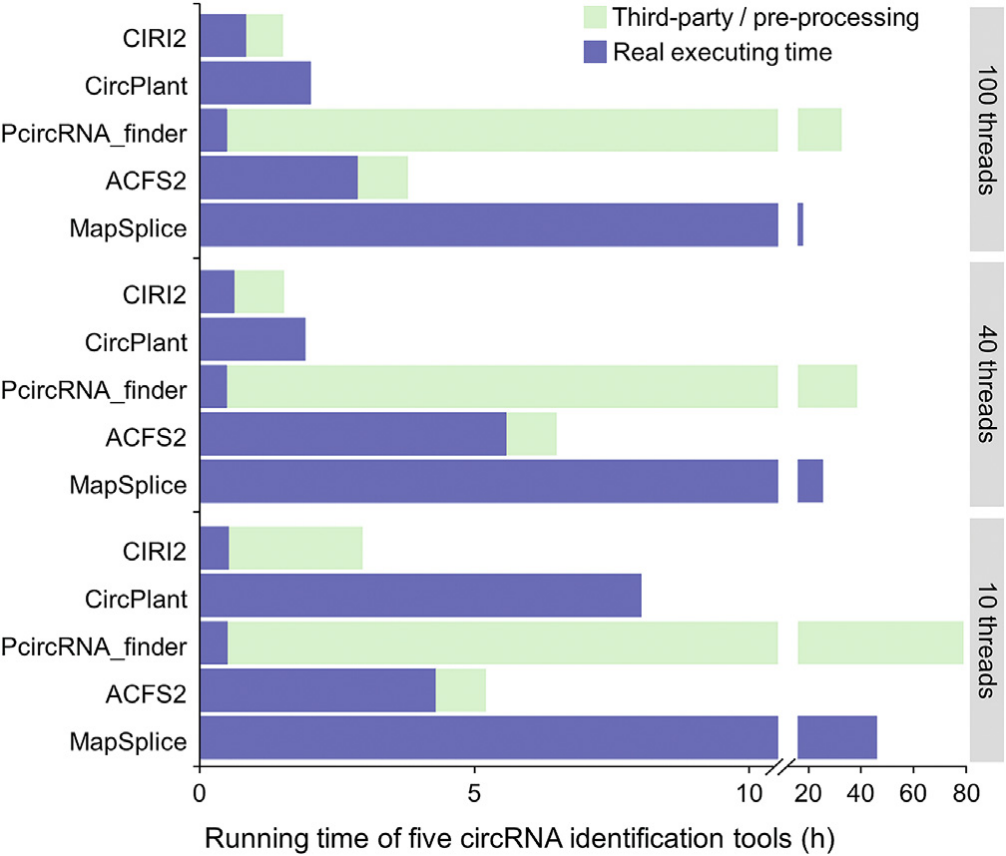
**Performance comparison of five circRNA detection tools** Five circRNA identification tools are tested on a same RNA-seq dataset. For each tool, the total executing time was separated into two parts: time for the dependencies (third-party or pre-processing programs needed by the tool) and time for the main algorithm (real executing time). The results are represented by stacked bars with different colors. MapSplice and CircPlant did not have executing time for third-party / pre-processing procedures, as their dependencies are integrated into their main routines. For procedures with parallel processing, only the longest executing time was counted.

CircPlant has several key differences in comparison to other circRNA detection tools. Firstly, CircPlant is a lightweight tool with fewer dependencies (Table 1). Except for the read mapping tool BWA-MEM, no other software or package is required to use CircPlant, which allows for a more straightforward installation. Secondly, CircPlant is memory- and time-efficient. We tested five prediction tools on a real paired-end RNA-seq dataset (ERR748773; paired-end, 100 bp, 123,120,011 × 2 reads), and CircPlant had a higher read mapping rate (Table S1) with relatively short computation time (Figure 2 and Table S2). This high efficiency will provide a considerable advantage for working with large-scale datasets. Thirdly, CircPlant applies several procedures to increase the sensitivity and precision of circRNA identification. Raw reads are mapped to a pseudoRef that is created with chiastic back-splice site flanking sequences. For paired-end datasets, candidate circRNAs should have back-splice reads in inferred regions of both paired reads, and that paired-end information would be consistent with corresponding templates of putative circRNAs. Maximum likelihood estimation based on multiple seed matching was adapted in CIRI2, resulting in the paired-end mapping information being consistent with stricter criteria in length, alignment score, and mismatch numbers to filter false positives derived from repetitive sequences and mapping errors [45]. It is recommended to use genome annotation and splicing signal at the same time, as they could be complementary to each other. Although exon–intron boundary usually has a canonical splicing signal, it is better to harness splicing signals in complete genome annotations for non-model species, usually with GT-AG [46], [47].

**Supplementary figure 1:**
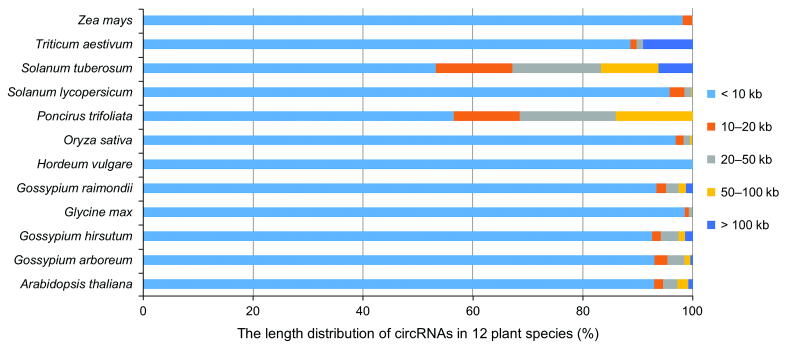
**The length distribution of circRNAs in PlantCircBase.**

To better detect plant circRNAs, CircPlant has considered the differences between plant and mammal genomes, and applies several plant-specific criteria for circRNA identification. (1) According to the length distribution of circRNAs belonging to 12 plant species in PlantCircBase [48], the default length threshold of circRNA is set to 20 kb, and the maximum threshold is set to 100 kb (Figure S1). (2) In the read mapping process, CircPlant applies stricter criteria, including alignment score and mismatch numbers, to exclude some false predictions due to high copy number of genes, many rounds of segment duplications, and whole-genome duplications in plant genomes. Genome annotations are extremely recommended to use for plant circRNA identification. (3) 5-bp flanking regions of the two canonical back-splice sites (acceptor and donor) are allowed for candidate back-splice sites in CircPlant [30]. circRNAs with minor changes in alternative donor/acceptor sites are another form of alternative circulation [25], while much of alternative splicing of circRNAs occurs nearby canonical splicing sites. (4) Besides the splicing by U2 based spliceosome (usually with consensus sequence GT-AG and GC-AG), CircPlant also considers a U12 based minor spliceosome (usually with consensus sequence AT-AC) for each candidate circRNA’s splicing signal. These two types account for over 99.5% of splicing sites [47]. These plant-specific features are integrated into CIRI2 for back-splice site collection.

### Benchmark based on simulated and real datasets

In order to fully gauge the performance of CircPlant, we compared it to four popular circRNA detection tools (MapSplice, ACFS2, PcircRNA_finder, and CIRI2) using both simulated and real datasets [30]. MapSplice is a highly accurate algorithm for detecting novel canonical and non-canonical splices, and is not dependent on splice site features or intron length [26]. ACFS allows *de novo*, accurate, and fast identification and abundance quantification of circRNAs from RNA-seq data, which is used more in human circRNA identification [28]. ACFS version 2 (ACFS2) was used in this study. PcircRNA_finder is the first precise prediction method for plant exonic circRNAs [30], and CIRI2 is a widely used tool for circRNA identification based on multiple seed matching [24].

**Supplementary figure 2:**
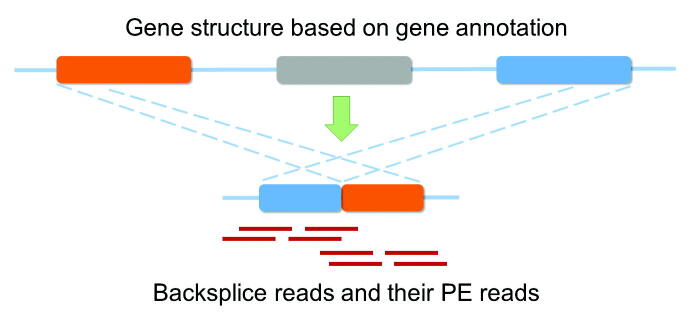
**Generation of simulated back-splice reads.** A simulated back-splice read generation process is shown in this figure. The orange and blue tiles denote two randomly chosen exons in a gene, and the red line segments denote generated reads. PE, paired end.

Two simulated RNA-seq datasets (paired-end reads, 100 bp, and 6000 back-splice reads for each sample; Figure S2) were generated by randomly choosing 200 chiastic transcripts based upon the *Arabidopsis thaliana* (TAIR10) and *Oryza sativa* (IRGSP-1.0.38) genome annotations, respectively. Sensitivity, precision, and F1 score (the harmonic mean of precision and sensitivity) were used to evaluate the performance of these methods [49]. The results indicated that CircPlant had the highest overall sensitivity among all five tools, and its precision reached 0.99 for both test genomes (Table 2). Considering that the ACFS2 algorithm contains over 95% of all canonical splicing sites in human transcriptome, it is not surprising that ACFS2 performed poorly with simulated datasets. Finally, CircPlant also obtained the highest F1 score in both test plant species compared to MapSplice, ACFS2, PcircRNA_finder, and CIRI2 (Table 2).

**Table 2:** **Evaluation of five different circRNA detection methods based on simulated datasets in *A. thaliana* and *O. sativa***

Species	Software	Sensitivity	Precision	F1 score
*A. thaliana*	MapSplice	0.30	0.91	0.45
	ACFS2	0.01	0.33	0.01
	PcircRNA_finder	0.85	0.90	0.87
	CircPlant	0.92	0.99	0.95
	CIRI2	0.88	0.95	0.91
*O. sativa*	MapSplice	0.26	0.76	0.39
	ACFS2	0.00	/	0.00
	PcircRNA_finder	0.69	0.92	0.79
	CircPlant	0.96	0.99	0.98
	CIRI2	0.96	0.99	0.97

*Note*: Sensitivity = TP/(TP + FN); Precision = TP/(TP + FP); F1 score = 2 × TP/(2 × TP + FP + FN), in which TP (true positive), FP (false positive), and FN (false negative) represent the number of correctly identified circRNAs, the number of incorrectly identified circRNAs, and the number of missing circRNAs, respectively. Sensitivity, precision, and F1 score all range from 0 to 1; higher value means higher accuracy and balance.

**Supplementary figure 3:**
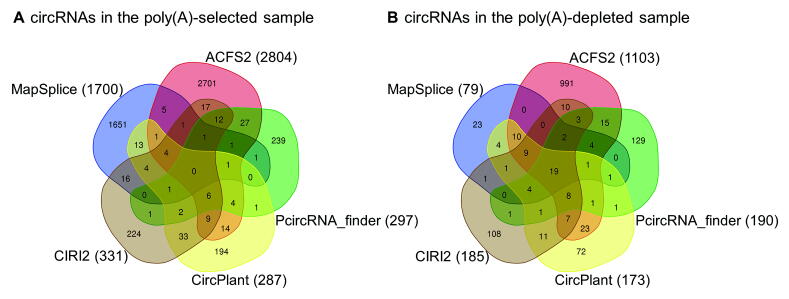
**Numbers of circRNAs predicted by five methods.** Two *Oryza sativa* RNA-seq datasets were used to evaluate the circRNA identification capability of CircPlant and other existing tools on poly(A)-selected (**A**) and poly(A)-depleted samples (**B**).

To test the circRNA detection capability of current tools on real datasets, we have obtained poly(A)-selected (ERR748773; paired-end, 100 bp, 123,120,011 × 2 reads) and poly(A)-depleted (ERR748783; paired-end, 100 bp, 207,396,249 × 2 reads) samples from mature leaves of *O. sativa*. These data were used to predict circRNAs by all five circRNA detection methods. In the poly(A)-selected sample, 287 circRNAs were predicted by CircPlant, while 1700 circRNAs were predicted by MapSplice, 2804 by ACFS2, 297 by PcircRNA_finder, and 331 by CIRI2 (Figure S3A). In the poly(A)-depleted sample, 173 circRNAs were predicted by CircPlant, while 79 circRNAs were predicted by MapSplice, 1103 by ACFS2, 190 by PcircRNA_finder, and 185 by CIRI2 (Figure S3B). Considering the secondary structure of circRNAs, RNA-seq of poly(A)-selected samples preferentially detects linear but not circular RNAs [50]. CircPlant predicted fewer circRNAs in the poly(A)-selected sample than the other four tools. However, in the poly(A)-depleted sample, CircPlant predicted more circRNAs than MapSplice and slightly less circRNAs than CIRI2 and PcircRNA_finder. Meanwhile, the circRNAs predicted by CircPlant exhibited relatively high levels of overlap with the circRNAs predicted by other tools (32.4% for poly(A)-selected sample and 58.4% for poly(A)-depleted sample). It suggested that CircPlant performs well in identifying the circRNAs.

Although PcircRNA_finder and CircPlant are both specifically developed for plant circRNAs, the overlapping circRNAs of these two tools in the poly(A)-selected and poly(A)-depleted samples are few, while the majority of circRNAs detected by CircPlant are overlapped with other tools (Figure S3). The limitations of PcircRNA_finder could be an important reason, as it is only able to detect exonic circRNAs. Interestingly, PcircRNA_finder uses the integration of five software results, including Bowtie used by MapSplice, but the overlap of these two tools is relatively low.

## Conclusion

Detection of circRNAs is the basis for in-depth study of circRNA’s biogenesis, functions, and corresponding molecular mechanisms. Considering the differences between plant and mammal genomes, most prevailing tools are unsuitable for plant circRNA detection. Computational requirements are also a concern when the identification process is scaled up to a large set of samples. CircPlant is a reliable, time-saving, and easy-to-use tool for plant circRNA detection and functional prediction. The CircPlant package is developed in Perl language, which has been tested on Ubuntu Linux 14.04. The software package and a user manual can be obtained from http://bis.zju.edu.cn/circplant.

## Code availability

CircPlant is freely available at http://bis.zju.edu.cn/circplant.

## CRediT author statement

**Peijing Zhang:** Conceptualization, Software, Formal analysis, Writing - original draft, Writing - review & editing. **Yongjing Liu:** Writing - original draft, Writing - review & editing. **Hongjun Chen:** Software. **Xianwen Meng:** Formal analysis. **Jitong Xue:** Software. **Kunsong Chen:** Writing - review & editing. **Ming Chen:** Conceptualization, Writing - original draft, Writing - review & editing, Supervision. All authors read and approved the final manuscript.

## Competing interests

The authors have declared no competing interests.
