# Supplementary material for: CircPlant: An Integrated Tool for circRNA Detection and Functional Prediction in Plants
Source: Genomics Proteomics Bioinformatics. 2020 Nov 4;18(3):352–8. doi: 10.1016/j.gpb.2020.10.001 (PMC7801249; doi:10.1016/j.gpb.2020.10.001)
Supplement: Supplementary data 1 [file mmc1.docx]

**File S1 Method of CircPlant**

CircPlant contains four modules, including circRNA detection, circRNA-miRNA interaction prediction, circRNA-miRNA-mRNA network construction, and circRNA annotation.

**circRNA detection**

The sequence, genomic location, and full length of circRNAs will be obtained by the first module, which is the core part of CircPlant. After raw reads are aligned to the reference genome by BWA-MEM, the modified CIRI2 is applied to collect as many back-splice sites as possible. After that, three processes can be performed to prioritize candidate circRNAs among those sites.

(1) If annotation file is provided, CircPlant would annotate candidate back-splice sites by comparing those sites to exon-intron boundary with gene annotation. circRNA was verified to be consistent with exon boundary in rice [33] which suggested that circRNAs share the same splicing sites with pre-mRNA splicing. And biogenesis of circRNA is dependent on canonical spliceosome machinery even though circRNA’s back-splicing site is cryptic and flexible [37]. These analyses insinuated that circRNA is the product from another form of alternative splicing.

(2) CircPlant creates a pseudoRef with chiastic back-splice site flanking sequences and then maps raw reads to it to check the back-splice sites. This process is referred to as second alignment.

(3) The third process is specifically designed for paired-end dataset. If candidate circRNAs have back-splice reads from one paired read, the other paired read should locate in an inferred region. Besides back-splice reads should be aligned to the same chromosome but in opposite orientation [27], those pairs have to be consistent with the corresponding template of putative circRNA [29].

Two files, ‘circRNA.circ’ and 'isoforms.circ', would be generated after these processes. The file ‘circRNA.circ’ contains specific information of back-splice sites, while 'isoforms.circ' contains full-length circRNAs as well as their isoforms in FASTA format. For exonic circRNAs generated from exons of a single protein-coding gene, CircPlant only extracts the exon regions based on genome annotation, and circRNA isoforms resulting from different transcripts are also included.

**circRNA–miRNA interaction prediction**

The second module allows the prediction of potential interactions between circRNAs and miRNAs. A two-step prediction is applied in this module, and the identified interactions will be stored in the output file ‘circRNA-miRNA.circ’.

(1) The first step aims to identify targets perfectly bound by miRNAs. A miRNA is related to the circRNA if its target sequence is discovered in the circRNA sequence. The target sequences of all known miRNAs are predicted by TargetFinder, with a strict score threshold no more than three.

(2) To identify the potential target mimics which are undetectable by traditional tools, Tapir is used in this step with three filtering rules: (a) the bulge in the complementary site should be composed only of three sequential nucleotides and located in the middle of the corresponding miRNAs. The middle position is defined as ninth to 10th, 10th to 11th, or 11th to 12th. (b) Perfect pairing is required from the second position to the bulge starting position including G/U pairs at the miRNA 5ʹ end. (c) Except for the central bulge, for a given mimic target and the miRNA pairing regions, the total mismatches (including G/U pairs) should be no more than three and the consecutive mismatches should not exceed two [42,43].

**circRNA–miRNA–mRNA network construction**

The third module is to get ceRNA pairs and circRNA–miRNA–mRNA networks.

After obtaining the interactions between known miRNAs and mRNAs using TargetFinder, the file ‘cepair.circ’ that contains a ceRNA pair list would be generated. The ceRNA pair is defined by a three-step procedure. For a circRNA ‘X’ and a mRNA ‘M’, (1) identifying all miRNAs that target transcript ‘M’ from the results of TargetFinder, (2) identifying all miRNAs that could bind circRNA ‘X’ from the results of TargetFinder (perfect binding) and Tapir (potential mimics), and (3) if one or more miRNAs could bind to both ‘X’ and ‘M’, we defined circRNA ‘X’ as a ceRNA of transcript ‘M’ and they thus represent a ceRNA pair. Then, a hypergeometric test is executed for each pair separately to assess the reliability of ceRNA pair prediction.

The interactions among circRNAs, miRNAs and mRNAs from ceRNA pairs are stored in the 'circRNA-miRNA-mRNA.circ' file, which could be visualized as networks using Cytoscape.

**circRNA annotation**

The fourth module is to predict the potential biological functions of circRNAs by using Gene Ontology (GO) annotation method. The GO information of a successfully annotated circRNA would be written to a corresponding file in the ‘anno’ folder.

For ceRNA pairs, if different members are regulated by the same miRNA, they are more likely to have similar functions. Therefore, the potential functions of a circRNA are defined as the GO terms annotated to its partner mRNAs in the ceRNA pairs. Moreover, a Fisher’s exact test is used to evaluate the enrichment of GO terms in the gene sets.
